# Hepatic intra-arterial chemotherapy in patients with advanced primary liver tumours

**DOI:** 10.3332/ecancer.2012.280

**Published:** 2012-11-20

**Authors:** Francesca Spada, Nicola Fazio, Guido Bonomo, Lorenzo Monfardini, Paolo Della Vigna, Davide Radice, Sabrina Boselli, Franco Orsi

**Affiliations:** 1 Upper GI and NET Unit, Department of Medicine, European Institute of Oncology (IEO), Via Ripamonti 435, 20141 Milan, Italy; 2 Division of Interventional Radiology, European Institute of Oncology (IEO), Via Ripamonti 435, 20141 Milan, Italy; 3 Division of Epidemiology and Biostatistics, European Institute of Oncology (IEO), Via Ripamonti 435, 20141 Milan, Italy; 4 Department of Medicine, European Institute of Oncology (IEO), Via Ripamonti 435, 20141 Milan, Italy

**Keywords:** hepatic arterial chemotherapy, liver tumours, hepatocellular carcinoma, biliary tract carcinoma

## Abstract

**Background:**

Primary liver tumours (PLTs) are currently a major health problem worldwide. The study’s aim was to investigate the feasibility, toxicity, and activity of hepatic intra-arterial chemotherapy (HIAC) in patients with advanced PLTs.

**Methods:**

We retrospectively analysed 43 patients with advanced unresectable PLT, who were consecutively treated. HIAC with 5-fluorouracil, cisplatin, and mitomycin-C was administered through a radiologically positioned temporary percutaneous catheter every six weeks until tumour progression or unacceptable toxicity was reached.

**Results:**

Partial response was observed in 26% and stable disease in 41% of patients. The median overall survival was 12.3 months. Manageable catheter-related complications occurred in 23% of patients. The grade 3–4 toxicities included neutropenia, thrombocytopenia, and transaminitis. There were no toxic deaths.

**Conclusion:**

The results of this retrospective study show that HIAC is feasible, active, and manageable in patients with PLTs. The treatment could be studied in selected patients with advanced progressive HCC/BTC being treated with or ineligible for sorafenib/cisplatin plus gemcitabine.

## Introduction

Hepatocellular carcinomas (HCCs) account for approximately 90% of primary liver cancers, the remaining 10% are biliary tract carcinomas (BTCs). HCCs are the sixth most common cancer in terms of incidence [1] and the third most common cause of cancer death worldwide. The incidence and mortality rates in Italy have been decreasing since the 1970s probably because of the prevention of viral hepatitis B and C, and improved curative treatments such as liver transplantation, resection, and percutaneous ablation [2]. The prognosis and feasibility of treatment for HCC patients largely depend on the characteristics of the tumour and on the severity of the underlying chronic liver disease, which affects most of them.

Radical resection is the only potentially curative treatment for HCCs and BTCs but it is frequently not possible because of advanced intra-hepatic disease [3, 4]. Patients with unresectable tumours have a poor prognosis with a median overall survival (OS) of less than six months from the time of diagnosis, which is only marginally improved by ablative or systemic therapies [5, 6].

Before the advent of targeted molecular therapies, systemic chemotherapy with single agents or drug combinations (the most widely used drugs were fluorouracil, epirubicin and cisplatin) led to a less than 20% response rate (RR) and 2–6 months’ median OS [7]. However, there have recently been some changes in systemic treatment strategies for advanced HCC and BTC [8, 11]. Sorafenib, a targeted agent aimed at the vascular endothelial growth factor receptor and ras/raf/mitogen-activated protein kinase pathways, has become a standard first-line therapy on the basis of the results of a large randomised phase III study called Sorafenib HCC Assessment Randomized Protocol (SHARP trial) [12]. Furthermore, the recent results of a multicentre randomised phase III trial showing that the combination of gemcitabine plus cisplatin has advantages over gemcitabine alone, has defined a possible standard first-line systemic treatment for advanced BTC [13].

Given the modest results of systemic chemotherapy and the poor liver function of most patients with advanced HCC and BTC, attempts have been made to administer chemotherapeutics directly into the hepatic artery. It has been reported that repeated hepatic arterial infusion chemotherapy (HIAC) with various drugs is useful in patients with advanced primary liver cancers. The rationale behind HIAC is that most of the tumour’s blood supply comes from the hepatic artery, whereas normal hepatic tissue receives most of its blood from the portal vein.

The theoretical advantage of HIAC over systemic chemotherapy is pharmacologically explained by the concepts of a “first-pass effect” and “increased local concentration” [14–20]. This route of administration is theoretically capable of reaching high-cancer drug concentrations with less systemic toxicity.

When the present trial was designed, there was no standard medical treatment for advanced primary liver cancer patients, and chemotherapy had not provided any clear clinical benefit in terms of survival. We studied the anti-tumoural activity of cisplatin, fluorouracil, and mitomycin-C administered by means of HIAC through a temporary percutaneous catheter.

## Materials and methods

### Patients and samples

This is a retrospective study involving 43 patients with advanced HCC and BTC who underwent consecutively HIAC between July 1997 and June 2003. They were unsuitable for surgical resection, liver transplantation, or non-surgical interventions (such as percutaneous ethanol injection, radiofrequency ablation, or trans-catheter arterial chemoembolisation) due to the involvement of both hepatic lobes. The other eligibility criteria were an age of ≥18 years, histologically or cytologically confirmed HCC or BTC, Child-Pugh A or B liver dysfunction, and an Eastern Cooperative Oncology Group performance status of 0 or 1. The organ function requirements included haemoglobin levels of ≥10 g/dL; an absolute peripheral granulocyte count of ≥1,500 mm^3^ and a platelet count of ≥60,000 mm^3^; serum albumin, AST, ALT, total bilirubin and alkaline phosphatase levels ≤2.5 times above the institutional normal range; and a prothrombin time of ≤3 seconds more than the institutional normal range. The exclusion criteria were New York Heart Association class II or greater heart failure or uncontrolled arrhythmia; a history of significant gastrointestinal bleeding requiring intervention within the previous three months; oesophageal varix, and previous malignancy. Previous anti-tumour therapy was allowed, including surgery, trans-arterial chemoembolisation (TACE), one systemic therapy regimen, and external-beam radiotherapy.

The baseline evaluation included a complete medical history and physical examination; an abdominal computed tomography (CT), including the pelvis or thorax (if this was considered useful for disease staging); a chest X-ray (postero-anterior and lateral views); electrocardiography (ECG); a complete blood count, and serum total bilirubin, transaminases, gamma-glutamyl-transpherase (gamma-GT), alkaline phosphatase, albumin, creatinine, blood urea nitrogen (BUN), lactate-dehydrogenase, cholinesterase, and alfa-fetoprotein (AFP).

### Study requirements

Twenty days before starting treatment, the patients had their history taken and underwent a physical examination, complete blood count, blood chemistry (including liver function tests), kidney function tests, ECG, abdominal CT, chest X-Ray, and the measurement of prothrombin time and the levels of albumin, ammonium, glucose, AFP, lactate-dehydrogenase. All of the patients underwent blood tests every week for the first four weeks after starting treatment: a complete blood count and the measurement of bilirubin, transaminases, gamma-GT, alkaline phosphatase, and creatinine during the first two weeks; a complete blood count alone during the third and fourth week. All of the patients also underwent abdominal CT 4–6 weeks after starting treatment in order to evaluate treatment response.

Drug-related toxic effects were graded using the NCI common terminology criteria for adverse events (CTCAE) version 2.0 [21].

### Catheter placement

All of the patients underwent angiography of the celiac and superior mesenteric arteries in order to determine arterial blood supply to the liver, stomach, and duodenum. If they had a normal vascular anatomy, after the injection of a local anesthetic, a removable 2.5 F percutaneous catheter was inserted through the axillary or subclavian artery (preferably on the left) with its tip in the proper hepatic artery and its proximal end attached to the thorax. The catheter remained in place throughout each chemotherapy course.

Gastroduodenal (GD) and right gastric (RG) artery embolisation was not mandatory at the beginning of the study, but was permitted using one or more coils. In the case of patients with an anatomic vascular variant that did not allow a full hepatic blood supply, blood redistribution was obtained by coil embolising the abnormal arterial branches, and the distribution of arterial flow was verified by means of helical CT during the course of an intra-arterial injection of contrast medium. Once complete arterial liver perfusion was obtained, the GD artery was embolised, and the tip of the catheter was left in the main arterial branch for the liver, where it remained movable, whereas its proximal end was fixed to the thorax.

After catheterisation, the patients returned to the Medical Oncology Division, and drug infusion was started. During the following days, they underwent an abdominal X-ray in order to ensure that the catheter was correctly positioned. The catheter was removed on the third day.

Finally, a chest and abdominal CT was performed four weeks after every course of HIAC to verify disease progress.

### Treatment and dose modification

Immediately after the placement of the catheter, all of the patients received a continuous intrarterial (i.a.) infusion of 5-fluorouracil (5-FU) 1,000 mg/m^2^/day diluted in 1,000 cc of saline solution plus heparin 5,000 units (U) in 24 h over three days. Furthermore, every patient received 15-min i.a. boluses of cisplatin (CDDP) 10 mg/m^2^ and mitomycin C (MMC) 2 mg/m^2^ twice daily for three days. The cisplatin and MMC were diluted in 20 cc of saline solution and administered slowly with a 15-min interval between them. Antiemetic premedication with i.v. boluses of metoclopramide 10 mg diluted in 100 cc of saline solution and dexamethasone 8 mg diluted in 100 cc of saline solution was given twice daily before CDDP and MMC. The patients were hyperhydrated with 2,000 cc of saline solution continuously over three days and were given ranitidine 150 mg/day i.v. to prevent gastroduodenal injury.

In the case of acute abdominal pain, the continuous 5-FU infusion was replaced by 500 U of heparin diluted in 100 cc of saline solution.

The patients underwent an abdominal X-ray every morning during treatment to control the position of the catheter tip, and complete blood counts and blood chemistry tests to assess acute hematological toxicity. Immediately after the end of the drug infusion on the third day, the catheter was removed, and a compressive dressing was left in place for 3 h before the patients were discharged.

Dose adjustments were made on the basis of the toxicity observed during each treatment cycle. The dose of all three drugs was reduced by 25 or 50% in the case of a neutrophil nadir of <1,000/mm^3^ lasting more than seven days or associated with high fever (more than two peaks of >38.0°C within 24 h or one peak >38.5°C), or a platelet nadir of <100,000/mm^3^. In the case of diarrhea or mucositis, 5-FU was reduced by 25%. The following treatment cycle was reduced by 30% in the case of any repeated grade 2 or 3 toxicity during the preceding cycle. Treatment was delayed until total recovery was made from any grade 3 clinical toxicity. After which, treatment was resumed with all three drug doses reduced by 50%. Any grade 4 clinical toxicity (except alopecia) required patient withdrawal from the protocol.

All of the patients who complained of upper gastrointestinal symptoms, such as epigastric pain, heartburn, dyspepsia, severe vomiting or nausea, underwent further radiological and clinical assessment.

### Study assessments

The primary aims of this retrospective single-centre analysis were to assess anti-tumoural activity in terms of partial response (PR), the disease control rate (DCR, defined as the sum of PRs and stable disease [SD]), and OS, all of which were determined on the basis of the results in all of the eligible patients. The secondary endpoints were the time to progression (TTP) and treatment toxicity. Responses were defined on the basis of the Response Evaluation Criteria in Solid Tumours (RECIST, 2000) [22]. A complete response (CR) required the complete disappearance of all CT-detectable disease and a PR >30% reduction in the sum of the products of the greatest perpendicular diameters of all of the tumour nodules detected by means of CT. Stable disease required neither a PR nor progressive disease (PD), which was defined as an at least 20% increase in the sum of the longest diameter of the target lesions.

### Statistical analysis

The RRs were tabulated as counts and percentages with binomial exact 95% confidence intervals (CI). OS was defined as the time from the first day of HIAC to the date of PD or death due to any cause. TTP was defined as the time from the first day of treatment to the date of PD or disease-related death. Both OS and TTP were estimated using the Kaplan–Meier method, and the differences between strata were assessed using the log-rank test. All of the *P* values were two-sided and considered significant if they were <0.05. The 95% CIs of median survival were constructed using log-log transformation [23].

The duration of SD was defined as the time between the date of the first PR and the date of the first PD.

The prognostic factors affecting patient survival considered in the survival analysis were HCC vs. BTC, ECOG status, Child-Pugh score, tumour extension, previous treatment, gender, HCV and HBV status, and cirrhosis.

All of the statistical analyses were made using SAS 9.2 software (Cary, NC, USA).

## Results

### Patient characteristics

Forty-four patients were consecutively treated between July 1997 and June 2003 but, as the diagnosis of one patient was changed from cholangiocarcinoma to haemangioendotelioma after a review of the histological samples, only 43 patients were included in the analysis. Their baseline characteristics and tumours are summarized in Table 1.

Twenty-nine patients (67%) were affected by HCC, including five (29%) of 17 patients did not have a sure diagnosis of chronic hepatopathy or cirrhosis; 14 patients (33%) had a BTC. The main causes of chronic hepatopathy were HCV infection and excessive alcohol intake. Twenty-four (92%) of the 26 patients with chronic hepatopathy showed clinical, radiological, or histological signs of cirrhosis: 22 (92%) with HCCs and 2 with BTCs. Most of the HCCs were classified as Cancer of the Liver Italian Programme (CLIP) 1 and BCLC (Barcelona Clinic Liver Cancer (BCLC) B. Fourteen patients had >50% liver involvement.

A total of 100 HIAC cycles were administered, with a median of two per patient (range 1–4). Three patients (7%) had radiologically confirmed ascites at baseline and six (14%) portal thrombosis.

### Clinical efficacy

One patient experienced rapid clinic worsening just after the first HIAC cycle related to clinical disease progression of disease and so he was included in the PD group even without radiological restaging. In terms of clinical responses, there were 0/11/17/15 CR/PR/SD/ PD. Particularly, partial response was achieved by 11 patients (26%: 4/14 with BTCs [28%] and 7/28 with HCCs [25%]) and SD by 17 (41%: 7/14 with BTCs [50%] and 10/28 with HCCs [36%]); 15 patients (35%) experienced PD. The median duration of PR in nine patients was 7.2 months overall (95% CI 2.2–16.9). In 5/7 pre-treated responders, the median PR was 7.2 months (95% CI 2.2–19.4) and in four untreated responders, it was 8.2 months (95% CI 4.7,16.9). Seventeen of the 28 responders (PR+SD) were pre-treated. The previous treatments were surgery alone in two (7%), surgery plus local treatment in two (7%), surgery plus local treatment plus systemic chemotherapy (5-fluorouracil-based regimens) in three (11%), local treatment (radiofrequency or chemoembolisation) in eight (29%), and tamoxifen in two (7%).

**Figure 1:**
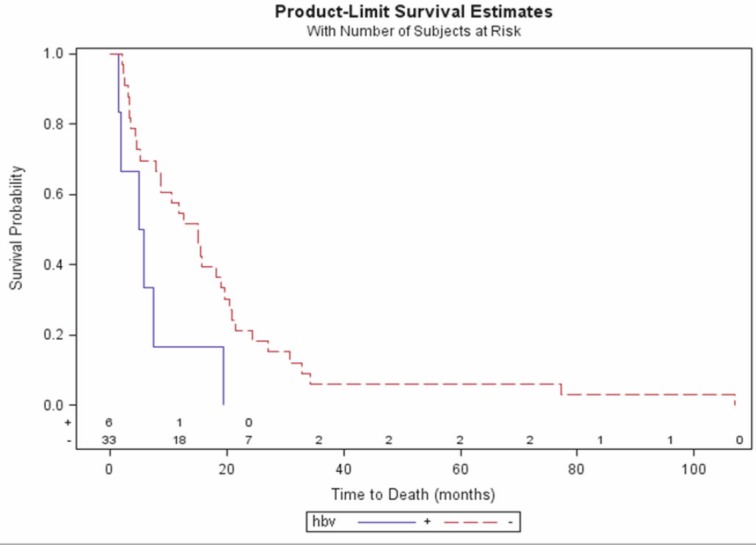
Overall survival by HBV status.

**Figure 2a:**
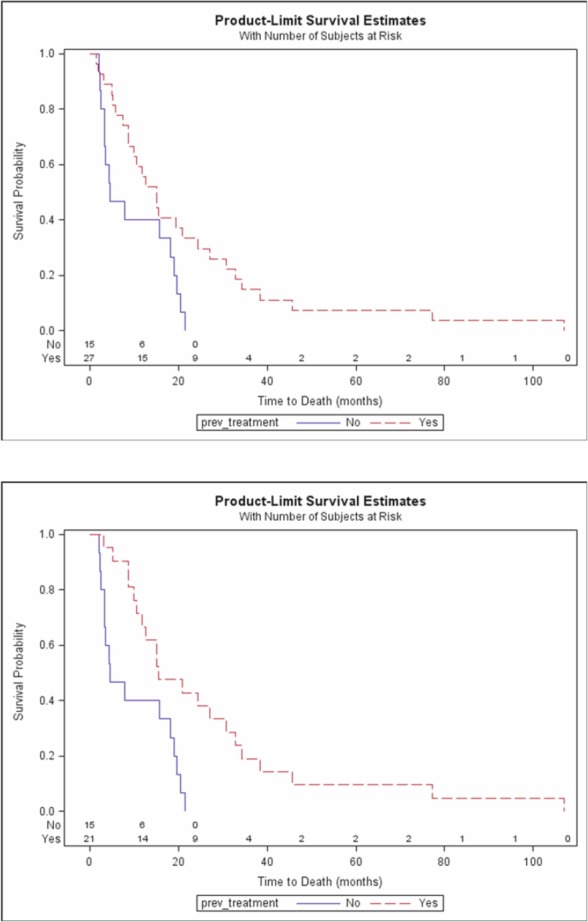
Overall survival by previous treatment. b: Overall survival by previous treatment for HBV negative patients.

Median OS was significantly longer in the HBV-negative patients than in those who were HBV positive (15 vs. 5.4 months; P = 0.024) (Figure 1), and there was a significant difference in OS between the pre-treated and the previously untreated patients (P = 0.018) (Figure 2a) and therefore also in the pre-treated HBV-negative patients than in the previously untreated HBV-negative patients (median 15.6 vs. 4.5 months; P = 0.002) (Figure 2b). TTP was also significantly longer in the HBV-negative patients (median 3.4 vs. 1.2 months; P = 0.024). By February 2010, only one of the 43 patients was still alive.

### Toxicity

Table 2 shows the main toxicities, which were generally mild/moderate and manageable. One patient developed febrile neutropenia, and two patients (5%) grade 3 thrombocytopenia without any clinical complications; seven patients (17%) experienced grade 3 increases in aminotransferase, alaninotransferase, and bilirubin levels. Grade 3 clinical toxicities occurred in <2% of the patients, and there were no grade 2–3 alopecia or neurology toxicities. One patient experienced hematemesis and melena after the first HIAC cycle. Two patients received HIAC with CDDP and MMC because of baseline thrombocytopenia. MMC was dropped in 10 cycles received by seven patients (16%). The dose of one or more drugs was reduced by 25 or 50% in nine patients (21%) because of haematological toxicity, mainly reduced platelet levels. There were no toxic deaths.

Catheter-related complications were evaluable in relation to 100 courses. The catheter was successfully implanted in all of the patients, with the sub-clavian route being used in 32 (74%). The distal end of the catheter was positioned in the proper hepatic artery in 27 patients (63%). Ten patients (23%) experienced temporary catheter-related complications: four gastric ulcers (9%), three catheter dislocations without replacement (7%), one thrombosis (2%), one catheter dislocation plus replacement (2%), and one catheter dislocation/replacement plus thrombosis (2%). The ulcers were treated with proton pump inhibitors (PPIs), sucralfate, and minor analgesics. All of the ulcers healed without complications. There was no case of catheter-related infection or severe bleeding (Table 3).

## Discussion

This retrospective analysis showed that HIAC with 5-FU, CDDP and MMC administered through a radiologically positioned temporary percutaneous catheter is feasible, active, and manageable therapy in patients with advanced HCCs or BTCs.

Doxorubicin, epirubicin, CDDP, 5-FU, MMC and etoposide (alone or in combination) have all been found to be active in patients with advanced HCC and/or BTC, with a ≤20% RR (CR+PR) in the case of HCCs,[24–27] and a 25-32% RR (CR+PR) in the case of BTCs.[28–32] However, no benefit in terms of OS has been observed in patients with HCCs, although the first evidence of a survival benefit has been reported in BTC patients.[14]

Liver-directed chemotherapy is an attractive option because it allows continuous infusion directly into the arterial bed that provide nearly all of the tumours’ blood supply. Over the last 15 years, many authors using HIAC with different drugs, schedules and pump systems have obtained high RRs with manageable toxicity. Most of these have used a CDDP- and 5-FU-based schedule, [18, 19, 33, 34] but there is still no generally accepted agreement concerning the most useful agents, or their optimal schedule, dose and treatment duration.

We used a high-dose, three-drug schedule given for shorter cycles; it was associated with a 67% higher DCR than those obtained using other schedules, [35] with the exception of the 88% DCR obtained by administering floxuridine and dexamethasone and TTP of seven months.[36]

Like those of most previous studies, our population consisted of patients with HCCs or BTCs, and we found that the DCR in the latter was higher than in the former (78% vs61%), thus suggesting that BTCs may be more sensitive to HIAC. Moreover, it is possible that a three-drug HIAC regimen is more synergistically effective than a two-drug regimen without any difference in toxicity.

Kemeny used a pump laparotomically positioned in a subcutaneous pocket on the abdominal wall to give a continuous HIAC infusion for 14 days.[37] We avoided the need for laparotomy. In spite of this, the presence of a permanent delivery system is not devoid of complications, estimated as 22-35%.[38] Thus, given that only some patients respond to therapy and many experience deteriorated liver function after HIAC, the real clinical benefit of implanting a permanent reservoir is questionable. Our patients were compliant to the therapy especially because of the relatively long-term treatment and hospitalisation,and the interval between cycles. However, as this was a retrospective analysis, we could not measure the QOL by means of a predetermined questionnaire.

In our study, only ten patients actually experienced catheter-related complications and these did not lead to any case of infection or severe bleeding. Nevertheless, HIAC was uncomfortable because of the need for admission to hospital and the unnatural position of the arm on the side in which the catheter was placed. Moreover, the radiological controls involved higher operating costs although our treatment was less expensive than pump infusion through a surgical catheter.

The adverse reactions of HIAC were tolerable and successfully managed by conservative treatment.

Median OS was 12.3 months with a slight but not statistically significant advantage in favour of the patients with BTC (19.1 vs 10.6 months). The analysed characteristics did not affect patient survival significantly, although previous treatment and being HBV-negative proved to be favourable prognostic factors. The favourable influence of previous treatment seems to correlate with the time between diagnosis and the beginning of HIAC, and may be explained by the fact that the earlier staged and less aggressive liver disease at diagnosis (in comparison with the previously untreated patients) allowed it to be successfully controlled by previous treatment.

In relation to the fact that the patients with a longer OS were HBV negative, it is well known that chemotherapy loses its efficacy because of the frequently observed p53 tumour suppressor gene mutations [39, 40] that contribute to the chemotherapy resistance of HCCs. Some proteins, such us X protein, capable of modulating apoptosis cell proliferation and the response to DNA damage, can also bind some p53 gene sequences, thus damaging tumour suppressor gene activity and facilitating carcinogenesis.[41,42]

It is worth noting that our population included 17 with radiological signs of liver disease but no histological or biochemical diagnosis, five of whom had HCCs. This is a significant finding because the etiology of HCCs throughout the world is mainly related to hepatitis viruses or alcohol. Although infrequent, the possibility that tumours develop in a healthy liver can be considered further proof of the potential relationship between HBV and carcinogenesis.

One limitation of our study is the heterogeneous nature of the population in terms of histology, etiology, tumour extension, previous treatment, and the presence of hepatopathy and/or ascites. It also included six patients with portal vein thrombosis, although this is often considered a contraindication for local treatment because of the reduced blood supply.

Another limitation of our analysis is the fact that the small sample size may have contributed to a lack of power in comparing the toxicity, efficacy and prognostic value of different chemotherapeutic regimens. However, the high DCR and the duration of the PRs and OS period may justify considering HIAC in particular subsets of patients with advanced or unresectable HCCs or BTCs.

In conclusion, although the lack of large-scale randomised studies, our findings demonstrate the feasibility, manageability and activity of repeated HIAC with 5-FU, CDDP and MMC in selected patients with unresectable advanced HCCs or BTCs. Given the absence of a standard second-line therapy for HCCs and BTCs, it could be considered in HCC patients who are refractory to/or progress on sorafenib, and in patients with liver-dominant metastatic BTCs who are refractory to/or progress on cisplatin plus gemcitabine.

The authors have no conflict of interest to declare.

## Figures and Tables

**Table 1.** Baseline patient and tumour characteristics.

No. of patients	43
HCC	29
BTC	14
Median age, years (range)	62 (43 – 69)
M/F	26/17
Performance status (ECOG) 0/1	31/12
Chronic liver disease, Yes/No	26/17
Etiology, HBV/HCV/HBV+HCV/Other	4/10/1/11
Cirrhosis	24
HCC	22
BTC	2
HCC Child-Pugh Class A/B^a^	20/2
BTC Child-Pugh Class A/B^a^	2/0
CLIP 0/1 – 3/4 – 6/NA	3/18/0/1
BCLC A/B/C/D/NA	5/17/0/0/0
Liver involvement <50%/≥50%	29/14
Lobar involvement	
Unilobar/bilobar	20/23
Distant metastases	3
Portal vein thrombosis	6
Ascites	3
Previous treatment (TACE/surgery/RFA/CT)	
Yes/No	27/16
*HCC* hepatocellular carcinoma cancer; *BTC* biliary tract cancer; *ECOG* Eastern Cooperative Group; *HBV* hepatitis B virus; *HCV* hepatitis C virus; *TACE* transcatheter arterial chemoembolisation; *RFA* radiofrequency ablation; *CT* chemotherapy; *NA* not available a Cirrhotic patients only	

**Table 2.** Toxicity.

Maximum NCI-CTC toxicity grade per patient				
Grade	1	2	3	4
WBC	5 (12%)	0	1 (2%)	1 (2%)
Neutrophils	2 (5%)	1 (2%)	1 (2%)	1^a^ (2%)
Hemoglobin	20 (47%)	3 (7%)	0	0
Platelets	19 (44%)	3 (7%)	2 (5%)	0
Ast/ALT	17 (40%)	9 (21%)	5 (12%)	0
Bilirubinemia	11 (26%)	2 (52%)	2 (5%)	0
Nausea/Vomiting	14 (33%)	5 (12%)	1 (2%)	0
Diarrhea	3 (7%)	0	1 (2%)	0
Mucositis	1 (2%)	3 (7%)	0	0
Asthenia	9 (21%)	8 (19%)	1 (2%)	0
Alopecia	2 (5%)	0	0	0
Abdominal pain	23 (54%)	4 (9%)	1 (2%)	0
Constipation	5 (12%)	1 (2%)	0	0
Neurology	7 (16%)	0	0	0
Fever	4 (9%)	2 (5%)	0	0
Haematemesis	0	0	0	1 (2%)

*NCI-CTC* National Cancer Institute Common Toxicity Criteria

a Febrile neutropenia

**Table 3.** Catheter-related complications.

Type of complicationt	No. of patients	%
Ulcer	4	9
Dislocation	3	7
Dislocation + repositioning	1	2
Dislocation + repositioning + thrombosis	1	2
Thrombosis	1	2
